# Dolutegravir plus lamivudine versus efavirenz plus tenofovir disoproxil fumarate and lamivudine in antiretroviral-naive adults with HIV-1 infection

**DOI:** 10.1186/s12879-021-06991-y

**Published:** 2022-01-04

**Authors:** Lisi Deng, Chunna Li, Ping Chen, Xiaoqing Luo, Xinchun Zheng, Lanlan Zhou, Yi Zhou, Jinyu Xia, Zhongsi Hong

**Affiliations:** 1 Department of Infectious Diseases, the Fifth Affiliated Hospital, Sun Yat-Sen University, 52 East Meihua Road, Zhuhai, 519000 Guangdong China; 2 Center for Disease Control and Prevention, Zhuhai, 519000 China

**Keywords:** Dolutegravir, Lamivudine, Two-drug regimen, Naive, HIV

## Abstract

**Background:**

Concerns regarding potential toxicity and drug-drug interactions during long-term treatment with three-drug active antiretroviral therapy (ART) regimens have been attracting increasing attention. We aimed to evaluate the efficacy and safety of dolutegravir (DTG) plus lamivudine (3TC) in ART-naive adults in China.

**Methods:**

This prospective observational cohort study enrolled HIV-naive inpatients treated with DTG + 3TC (2DR arm) or efavirenz (EFV) plus tenofovir disoproxil fumarate (TDF) and 3TC (3DR arm). There were no limits on baseline viral load. Inflammatory biomarkers were also investigated in the 2DR arm.

**Results:**

Between September 2019 and January 2020, 27 patients treated with DTG + 3TC and 28 patients treated with EFV + TDF + 3TC were enrolled in the study. At week 12, the proportion of patients with viral loads < 50 copies/mL in the 2DR arm was 81.5% (22/27) compared with 53.6% (15/28) in the 3DR arm (p < 0.01). At week 24, the proportion of patients with viral loads < 50 copies/mL in the 2DR arm was 100% (26/26) compared with 83.3% (20/24) in the 3DR arm (p < 0.05). Mean changes in CD4 cell counts from baseline at week 12 were 125.46 cells/µL in the 2DR arm and 41.20 cells/µL in the 3DR arm (p < 0.05). Mean changes in CD4 cell counts from baseline at week 24 were 209.68 cells/µL in the 2DR arm and 73.28 cells/µL in the 3DR arm (p < 0.05).

**Conclusions:**

DTG + 3TC achieved virologic suppression more rapidly than EFV + TDF + 3TC after 12 and 24 weeks. DTG + 3TC could represent an optimal regimen for advanced patients.

*Clinical Trial Registration* ChiCTR1900027640 (22/November/2019).

## Introduction

There has been a sustained decrease in the mortality and morbidity of patients with human immunodeficiency virus type 1 (HIV-1) infection since the introduction of highly active antiretroviral therapy (HAART) [1, 2]. However, with long-term use of HAART, problems can arise including adverse events (AEs) caused by antiretroviral drugs [3] and drug-drug interactions (DDIs) between HAART and other therapies for non-acquired immunodeficiency syndrome (AIDS)-related complications and anti-HIV-1 treatments [4]. Concerns related to the safety profiles of these medicines administered throughout the life course have attracted increasing attention. Specifically, patients with low CD4 + cell counts or high viral loads at baseline (prior to HAART) occur commonly in China. An optimized regimen that is well suited to such patients with advanced disease is needed.

Two-drug regimens (2DRs) have been investigated as a means to improve the quality of life of patients with HIV-1 by reducing adverse drug reactions, saving costs, and improving HAART compliance [5–7]. Dolutegravir (DTG), a second-generation integrase strand transfer inhibitor (INSTI), is attractive as a component of 2DRs because of its high antiviral potency and resistance barriers [8–10]. Lamivudine (3TC) is also an effective component of 2DRs with a well-documented safety profile and a high barrier to resistance [11].

In the GEMINI trials, the antiviral activity of DTG + 3TC was similar to that of DTG + TDF/emtricitabine (FTC) in ART-naive individuals with baseline viral loads below 500,000 copies/mL; furthermore, no resistance was reported for up to 96 weeks [11]. Antiviral regimens containing INSTIs were effective in preventing long-term mitochondrial, bone, and renal toxicity [14]. Thus, DTG + 3TC might represent a viable 2DR for the treatment of HIV-1 infection in ART-naive individuals.

Currently, international guidelines recommend DTG + 3TC as the first-line regimen for ART-naive individuals with HIV-1 infection and viral loads < 500,000 copies/mL. However, this regimen has not been evaluated in individuals with no limitations on viral loads and especially in individuals with baseline viral loads > 500,000 copies/mL. In addition, there is limited experience with DTG + 3TC in China.

Several potential mechanisms to explain the relationship between inflammation and HIV-1 replication have been proposed [15–17].One postulated mechanism for abnormal levels of inflammation and immune activation despite HAART is ongoing HIV-1 replication and/or expression of HIV-1 gene products [16]. Additionally, levels of inflammatory markers including interleukin (IL)-6, D-dimer, soluble tumor necrosis factor receptor-1, high-sensitivity C reactive protein (but not levels of T-cell activation, senescence, or exhaustion) are independently predictive of mortality in individuals with treated HIV-1 infection with a history of AIDS [18].

In this prospective cohort study, we aimed to evaluate the efficacy and safety of DTG + 3TC compared with a first line 3DR (EFV + TDF + 3TC) for treatment of HIV-1 infection in ART-naive adults in China with no limitations on baseline viral load.

## Methods

This ongoing observational single-center prospective observational cohort study enrolled ART-naive individuals with HIV-1 infection referred to our hospital from September 2019 to January 2020. Data were collected from September 2019 to July 2021. No CD4 count restrictions nor limitations on baseline viral loads were imposed.

Exclusion criteria included pre-existing major viral resistance mutations to nucleoside reverse transcriptase inhibitors, non-nucleoside reverse transcriptase inhibitors, or protease inhibitors; active hepatitis B virus infection; anticipated hepatitis C treatment during the study period; unstable or severe hepatic impairment; alcohol or drug abuse; pre-existing mental disorders; and pregnancy.

The study was approved by the Research Ethics Committee of the Fifth Affiliated Hospital of Sun Yat-sen University (No. ZDWY [2019], Lunzi No. K16-1). Written informed consent was obtained from each participant prior to the initiation of study procedures. All study procedures were performed in accordance with relevant local guidelines and regulations. The study was registered in the Chinese Clinical Trial Registry: ChiCTR1900027640 (22/November/2019).

### Procedures

HAART regimens were selected according to Chinese guidelines and patient requirements. For all patients, HAART regimens were selected on clinical grounds either because of concomitant diseases, the results of laboratory tests, financial reasons, AEs, or risk of DDIs.

Study visits were scheduled at baseline and weeks 2, 4, 8, 12, 24, 36, and 48 following HAART initiation. Renal function, liver enzymes, lipid profiles, blood counts, and bone mineral density were assessed at all study visits. Plasma HIV-1 RNA viral load was tested at weeks 12, 24, and 48 (Roche Diagnostics Limited, Shanghai, China). CD4 cell counts were determined at weeks 4, 12, 24, and 48. Levels of inflammatory biomarkers in individuals in the 2DR arm were assessed at baseline and at weeks 2, 4, and 12.

Safety, including AEs and serious AEs (SAEs), was assessed at each study visit. Events were graded according to the Division of AIDS Table for Grading the Severity of Adult and Pediatric Adverse Events, version 2.0 [19]. All SAEs and AEs of special interest were investigated until resolution, stabilization, loss of the patient to follow-up, or the event was otherwise explained.

### Outcomes

The primary efficacy endpoint was the proportion of participants with virologic success, defined as HIV-1 RNA viral load < 50 copies/mL. The secondary endpoints included evaluating the efficacy, safety, and tolerability of HAART regimens, assessing changes from baseline in CD4 + cell counts, and assessing changes from baseline in the CD4 + /CD8 + cell ratio. Inflammatory biomarkers were exploratory outcomes in the 2DR arm.

### Statistical analysis

χ^2^ and Fisher’s exact tests were used to assess differences between categorical variables. The student’s t-test or Wilcoxon’s signed-rank test were used to assess differences between continuous variables. Statistical analyses were performed using SPSS 22.0 (IBM Corporation, Armonk, NY, USA) and GraphPad Prism 6.0 (GraphPad Software, Inc. La Jolla, CA, USA). Values of P < 0.05 were considered statistically significant. Multivariate logistic regression analysis was used to identify factors influencing the viral suppression rate at week 12.

## Results

**Fig. 1:**
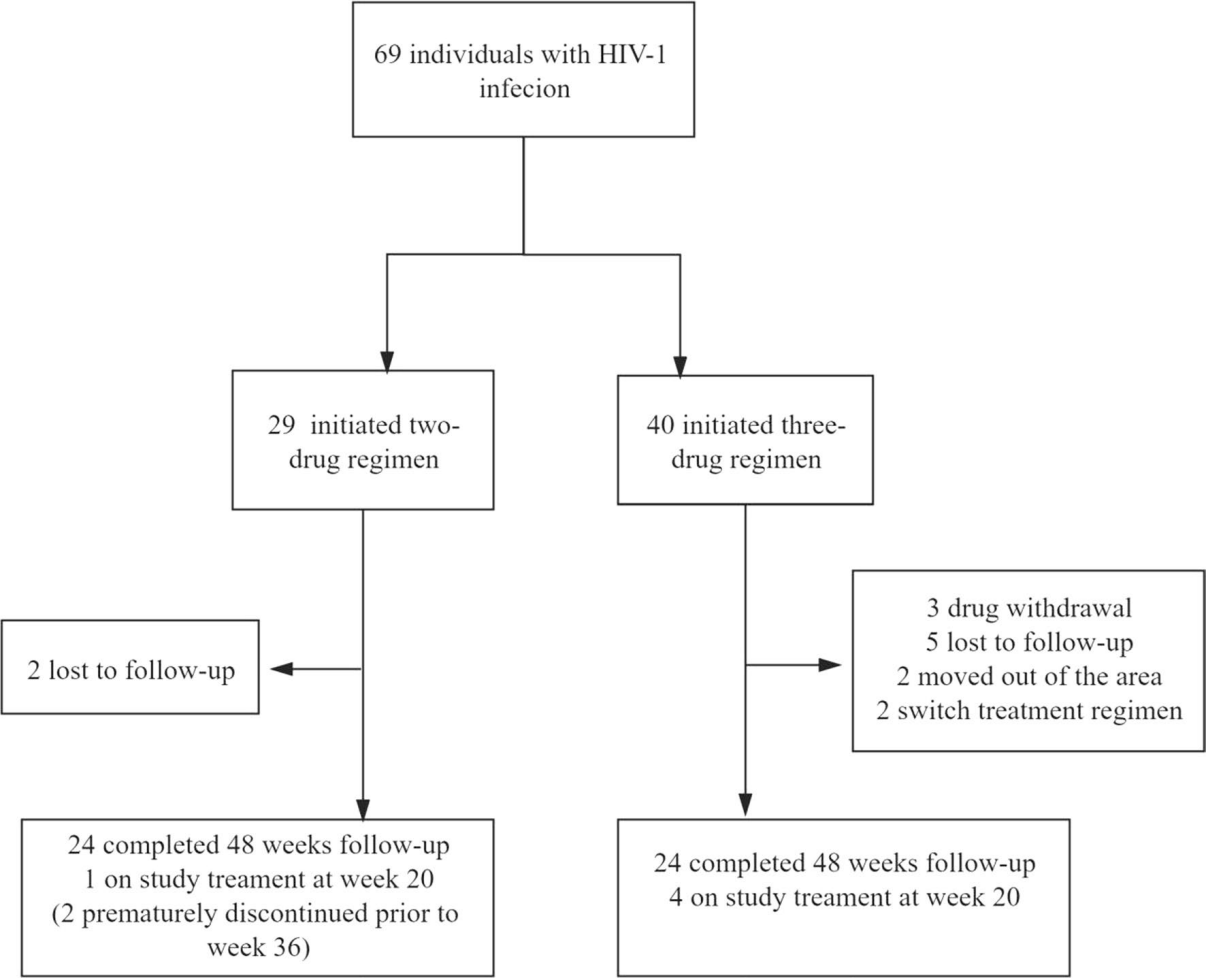
Study Profile

Between September 2, 2019 and January 26, 2020, 73 participants were screened and 69 were enrolled in this study. Genotypic tests were performed by the Chinese Center for Disease Control prior to HAART administration; none of the participants harbored viruses bearing mutations conferring resistance to INSTIs or NRTIs. In the 2DR group, two participants discontinued study follow-up prior to week 36 and were lost to follow-up. In the 3DR group, twelve participants discontinued study follow-up prior to week 8 for the following reasons: moved out of the area (n = 2), drug withdrawal (n = 3), lost to follow-up (n = 5), and change in HAART regimen (n = 2) (Fig. 1).

Baseline characteristics were generally similar between the two study arms with the exception that the proportions of participants with opportunistic infections and CD4 counts < 200 cells/µL were higher in the 2DR arm compared with the 3DR arm (Table 1). Participants were not included in the endpoint analysis if did not reach the follow-up time window. At week 12, the proportion of participants with HIV RNA < 50 copies/mL in the 2DR arm was 81.5% (22/27) compared with 53.6% (15/28) in the 3DR arm (p < 0.01) (Table 2). In the 2DR arm, 26 (100%) participants achieved virologic suppression at week 24 and maintained HIV-1 viral loads < 50 RNA copies/mL at week 48, despite the fact that nine (33.3%) participants had baseline HIV-1 RNA viral loads > 100,000 RNA copies per mL. In the 3DR arm, four (16.7%) participants did not achieve virologic success at week 24, with HIV-1 RNA levels between 50 and 200 copies/mL; however, all participants achieved virologic suppression at week 48. Virologic rebound had not observed in either study arm between week 24 and week 48. Multivariate logistic regression analysis indicated that none of age, opportunistic infections, CD4 cell counts < 200 cells/µL, and viral loads > 100,000 copies/mL were statistically significant determinants of viral suppression at week 12 (Table 3).

**Table 1:** Baseline demographics and clinical characteristics for the two study arms

Characteristic	2DR	3DR	P value
Age, y, median (range)	31 (24–38)	31 (24.25–40)	0.768
Male	27 (100%)	23 (82.14%)	0.051
Mode of transmission			
MSM	22 (81.48%)	20 (68.97%)	0.38
Heterosexual	5 (18.51%)	7 (25.00%)	0.561
IDU	0 (0%)	1 (3.57%)	1.000
Hepatitis B	0 (0%)	3 (10.71%)	0.236
Hepatitis C	0 (0%)	0 (0%)	–
Opportunistic infections	12 (44.44%)	4 (14.29%)	0.033
Pneumocystosis	5 (18.51%)	4 (14.29%)	0.954
Penicillium Malneffei	4 (14.81%)	0 (0%)	0.051
Cryptococcus	0 (0%)	1 (3.57%)	1.000
Candida	3 (11.11%)	1 (3.57%)	0.352
Tuberculosis	1 (3.7%)	0 (0%)	0.491
Cytomegalovirus	7 (25.93%)	2 (7.14%)	0.129
EB virus	4 (14.81%)	1 (3.57%)	0.305
Bacterial infection	4 (14.81%)	3 (10.71%)	0.916
CD4/CD8 ratio	0.25 ± 0.17	0.36 ± 0.19	0.031
CD4 + count (cells/μL)			
Mean	222.07 ± 176.67	326.55 ± 194.56	0.044
≤ 200 (cells/μL)	13 (48.15%)	5 (17.86%)	0.017
> 200 (cells/μL)	14 (51.85%)	22 (78.57%)	0.037
HIV-1 RNA ( copies/mL)			
Median (range)	61,100 (33,500–229,000)	42,600 (22,650–91,200)	0.167
≤ 100,000 (copies/mL)	18 (66.67%)	24 (85.71%)	0.179
> 100,000 (copies/mL)	9 (33.33%)	4 (14.29%)	0.179
ALT	17.7 (12–24)	20.45 (14.25–30.3)	0.210
AST	19 (17.5–25.2)	21.40 ± 5.14	0.705
Creatinine	75 (67–91)	77.14 ± 16.04	0.926
Cystatin	0.99 ± 0.38	0.89 (0.79–1)	0.076
Urea	3.94 (3.5–4.4)	4.19 ± 1.15	0.495
CHOL	3.79 ± 1.05	4.09 ± 1.18	0.328
HDL	0.99 (0.77–1.15)	1.06 ± 0.37	0.421
LDL	2.24 ± 0.91	2.60 ± 0.69	0.109
TG	131 ± 0.66	1.57 (0.7–1.96)	0.441
eGFR	100.56 ± 21.18	101.29 ± 14.23	0.905

Data are presented as mean (SD), medians (interquartile ranges) or No. (%)

IDU, intravenous drug user; MSM, men who have sex with men; ALT, alanine aminotransferase; AST, aspartate aminotransferase; CHOL,total cholesterol; LDL, low-density lipoprotein; HDL, high-density lipoprotein; TG, triglyceride; eGFR, estimated glomerular filtration rate; 2DR, two-drug regimen; 3DR, three-drug regimen

**Table 2:** Snapshot analysis of participants after ART treatment

Characteristic	2DR	3DR	P value
HIV-1 RNA (< 50 copies/mL)			
Week 12	25 (92.5%)	15 (53.57%)	0.003
Week 24	26 (100%)	20 (83.3%)	0.046
Week48	24 (100%)	24 (100%)	1.000
Change From Baseline in CD4 + count (cells/μL)			
Week 12	125.46 ± 149.38	41.2 ± 110.60	0.026
Week 24	209.68 ± 175.88	73.28 ± 162.40	0.020
Week48	204.73 ± 287.32	163.21 ± 121.15	0.805
Change From Baseline in CD4/CD8 ratio			
Week 12	0.11 (0.02–0.31)	0.21 (0.11–0.35)	0.058
Week 24	0.15 (0.09–0.36)	0.26 (0.12–0.49)	0.170
Week48	0.13 (0.07–0.39)	0.23 (0.10–0.61)	0.492
Change From Baseline to week 48 in Laboratory Results			
ALT	− 3.2 (− 4.55–1.1)	5 (− 0.85–16.35)	0.004
AST	− 4.38 ± 7.64	1.11 ± 10.41	0.217
Cystatin	− 0.1 ± 0.122	− 0.07 ± 0.10	0.439
Urea	− 0.01 ± 1.31	− 0.19 ± 1.13	0.716
eGFR	− 17.27 ± 24.87	2.84 (− 7.1–22.08)	0.052
Hypercholesterolemia	1 (3.70%)	2 (7.14%)	1
Hypertriglyceridemia	3 (11.11%)	5 (17.86%)	0.744
Elevated LDL	1 (3.70%)	0 (0%)	0.49
Elevated HDL	2 (7.41%)	8 (28.57%)	0.092
Elevated creatinine	3 (11.11%)	1 (3.57%)	0.577

Data are presented as mean (SD), medians (interquartile ranges) or No. (%)

Abbreviations: ALT, alanine aminotransferase; AST, aspartate aminotransferase; LDL, low-density lipoprotein; HDL, high-density lipoprotein; eGFR, estimated glomerular filtration rat; 2DR, two-drug regimen; 3DR, three-drug regimen

**Table 3:** Results of multivariate regression analysis

Variable	OR ( 95%CI)	P value
Age		
2DR	0.65 (0.25, 0.88)	0.11
3DR	1.03 (0.94, 1.14)	0.6
Combined with OIs		
2DR	1629 (NA, 1.10)	0.2
3DR	0.22 (0.00, 4.20)	0.3
CD4 cell counts < 200 cells/µL		
2DR	0.00 (0.00, 0.07)	0.9
3DR	1.01 (0.07, 24.7)	1.0
VL > 100,000 copies/mL		
2DR	0.01 (0.00, 16.6)	0.3
3DR	0.00	1.0

2DR, two-drug regimen; 3DR, three-drug regimen; OIs, opportunistic infections; VL, viral load

**Fig. 2:**
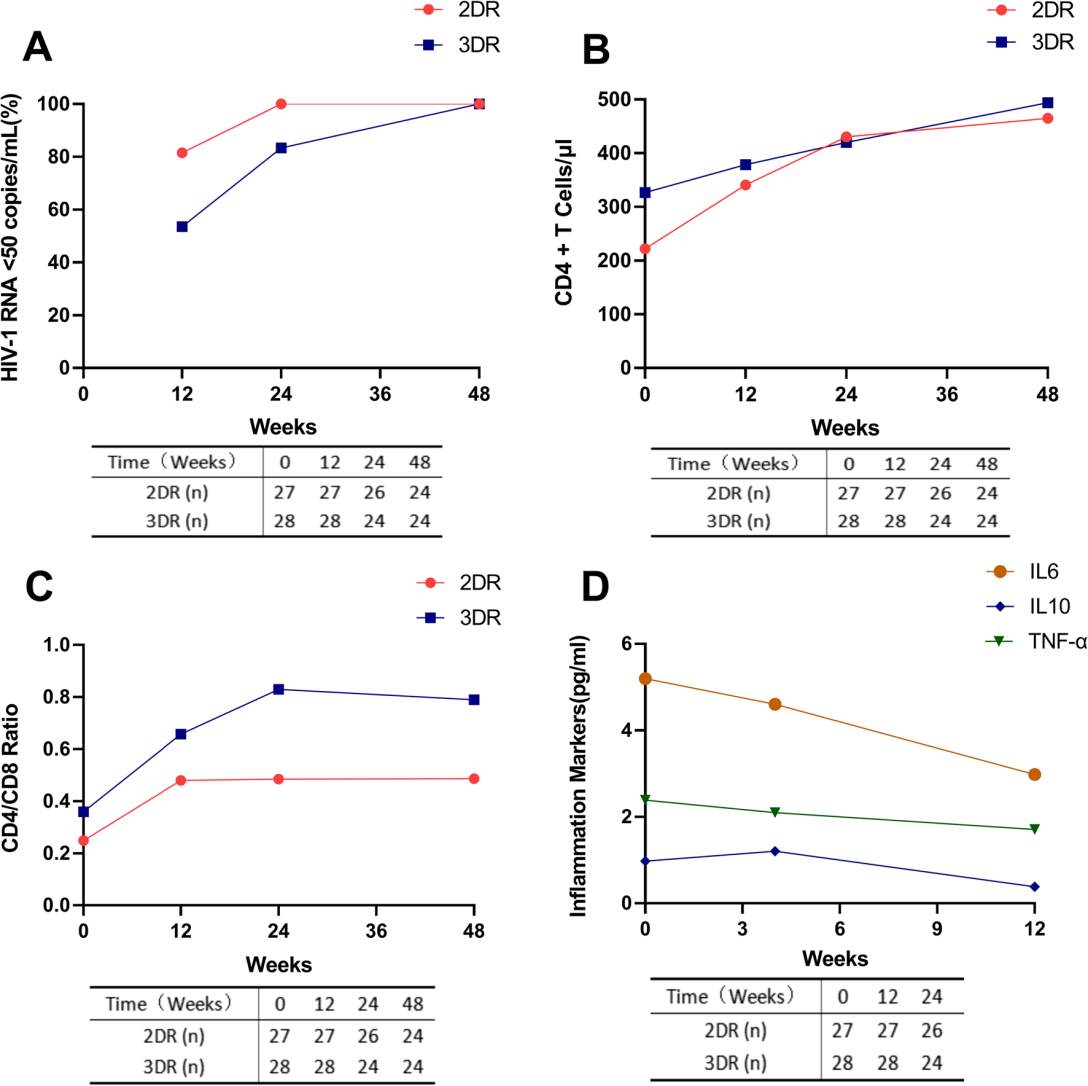
Dynamic changes in cellular and viral parameters as well as inflammatory markers in patients with HIV-1 infection treated with either a 2DR or a 3DR. Time-course parameters, including HIV-1 RNA viral load (**A**), peripheral CD4 + T cells (**B**), CD4/CD8 cell ratio (**C**), and inflammatory markers (IL-6, IL-10, and TNF-ɑ) (**D**), were recorded. Values of p < 0.05 were considered statistically significant. 2DR, two-drug regimen; 3DR, three-drug regimen; IL, interleukin; TNF, tumor necrosis factor

Mean baseline CD4 cell counts were 222.07 cells/µL in the 2DR arm and 326.55 cells/µL in the 3DR arm (p = 0.044). The mean baseline CD4/CD8 ratio was 0.25 in the 2DR arm and 0.36 in the 3DR arm (p = 0.031) (Table 1). Mean changes in CD4 counts from baseline to week 12 were 125.46 cells/µL in the 2DR arm and 41.20 cells/µL in the 3DR arm (p = 0.026). Mean changes in CD4 counts from baseline to week 24 were 209.68 cells/µL in the 2DR arm and 73.28 cells/µL in the 3DR arm (p = 0.020) (Table 2). Self-reported adherence was generally high throughout the study period in both arms. Elevated levels of inflammatory biomarkers including IL-6, IL-10, and tumor necrosis factor (TNF)-α were observed in patients with opportunistic infections in the 2DR arm at baseline. A rapid decline and return to normal levels of inflammatory biomarkers was observed by week 4 or week 12 (Fig. 2). Inflammatory biomarkers were not tested in the 3DR arm.

The most frequently reported AEs across both study arms were diarrhea, celialgia, emesis, nausea, fatigue, dizziness, rash, anxiety, and upper respiratory tract infection (Table 4). Numerically, fewer participants reported drug-related AEs in the 2DR arm than in the 3DR arm. There was a higher proportion of participants with dizziness in the 3DR arm (n = 15) than in the 2DR arm (n = 2) (p = 0.009). Drug-related AEs leading to drug withdrawal or a change in treatment regimen occurred in five participants in the 3DR arm (grade 4 hepatic damage, n = 2; grade 3/4 rash, n = 3) and in no participants in the 2DR arm.

**Table 4:** Adverse events overview

Adverse events	2DR	3DR	P value
Agrypnia^a^	0 (0%)	3 (10.71%)	0.236
Anxiety/depression^a^	1 (3.70%)	1 (3.5%)	1
Loss of appetite^a^	1 (3.70%)	3 (10.71%)	0.63
Diarrhea/celialgia^a^	1 (3.70%)	2 (7.14%)	0.97
Nausea^a^	1 (3.70%)	2 (7.14%)	0.97
Vomiting^a^	0 (0%)	5 (17.86%)	0.051
Fatigue^a^	4 (14.81%)	3 (10.71%)	1
Dizzy^a^	2 (7.41%)	15 (53.57%)	0.009
Upper respiratory tract infection^a^	4 (14.81%)	3 (10.71%)	1
Rash^a^	6 (22.22%)	9 (28.57%)	0.589
SAEs	0 (0%)	5 (15.15%)	0.058

SAE, serious adverse event; 2DR, two-drug regimen; 3DR, three-drug regimen

^a^All drug-related AEs were grade 2 or less

Elevated levels of alanine aminotransferase were more common in the 3DR arm than in the 2DR arm (p = 0.006) (Table 2). Increases in creatinine from baseline were observed in 11.11% (3/27) of participants in the 2DR arm and 3.5% (1/28) of participants in the 3DR arm (p = 0.577). No significant differences were observed between the 2DR and 3DR arms in mean changes from baseline in cystatin, urea, or estimated glomerular filtration rate (eGFR) at week 48. Elevated cholesterol, triglycerides, low density lipoprotein (LDL) were observed in both study arms. The proportions of participants with hypercholesterolemia, hypertriglyceridemia, and increased LDL were similar in both study arms.

## Discussion

In this ongoing prospective cohort study, we analyzed the efficacy and safety of DTG + 3TC compared with EFV + TDF + 3TC among ART-naive adults in China. DTG + 3TC was non-inferior to the standard 3DR (EFV + TDF + 3TC) through 48 weeks of treatment. Multivariate logistic regression analysis indicated that none of age, opportunistic infections, CD4 cell counts, and HIV-1 viral loads > 100,000 copies/mL were independently associated with treatment outcome. Importantly, the more rapid reduction in viral load at week 12 in the 2DR demonstrated that DTG + 3TC was statistically superior to the 3DR. In addition, increases in CD4 cell counts from baseline to week 12 and week 24 were more rapid in the 2DR arm than in the 3DR arm. The rapid reduction in viral load may be responsible for the immune reconstitution observed in the 2DR arm.

Data from the SINGLE study demonstrated the superior efficacy and better tolerability of DTG + abacavir + 3TC compared with EFV + TDF + FTC [20]. The GEMINI studies showed non-inferior virological efficacy of a 2DR (DTG + 3TC) compared with a recommended 3DR (DTG + TDF/FTC) in the first adequately powered, randomized, controlled studies of DTG + 3TC in HAART-naive patients with baseline viral loads of less than 500,000 copies/mL [12, 13]. Our study confirms key virological findings of the ACTG A5353 and PADDLE studies [6, 21]. In previous studies, 2DRs have been used as in treatment-experienced individuals with virologic suppression and showed safety and efficacy in maintaining viral suppression [38–41]. In recent years, data from real world studies have documented the feasibility and efficacy of 2DRs in ART-naive individuals [42–44].

Strikingly, both mean CD4 cell counts and CD4/CD8 ratios were lower in the 2DR arm compared with the 3DR arm at baseline. Low CD4 cell counts are strongly correlated with high HIV-1 RNA levels [22–24]. Low CD4/CD8 ratios are associated with increased risks of morbidity and mortality, and are prognostic of non-AIDS defining events in HIV-1-infected individuals [25–27]. The proportion of patients with opportunistic infections was higher in the 2DR arm compared with the 3DR arm. Numerically, the proportion of participants with viral loads above 100,000 RNA copies/mL was higher in the 2DR arm compared with the 3DR arm. Thus, the proportion of patients with advanced HIV-1 infection was higher in the 2DR arm.

There are continuing challenges in managing HIV-1 infection, particularly in older patients who often experience age-related comorbidities resulting in complex polypharmacy and increased risks of DDIs [28]. DTG has minimal drug interactions because its major and minor metabolic pathways are uridine 5′-diphospho-glucuronosyltransferase 1A1 and cytochrome 3A4, respectively [29]. In this study, the rate of virological suppression reached 92.5% at week 12 and 100% at week 24 in the 2DR arm. Taken together, the findings of this study demonstrated the superiority of a 2DR containing DTG in patients with advanced HIV-1 infection compared with the standard 3DR.

Increases in CD4 cell counts from baseline to week 12 and week 24 occurred more rapidly in the 2DR arm than in the 3DR arm. The recovery of CD4 cell counts reflects immune reconstitution. INSTIs, especially DTG and EVG, are associated with a higher probability of development of immune reconstitution inflammatory syndrome (IRIS) [30]. Inflammation is associated with untreated HIV-1 infection, and high baseline levels of IL-6, IL-10, and TNF-α are associated with increased risks of AIDS-defining events [31, 32]. In this study, elevated levels of inflammatory biomarkers including IL-6, IL-10, and TNF- α in the 2DR arm declined rapidly and returned to normal levels by week 4 or week 12. There were no signs of IRIS in patients with advanced HIV-1 in the 2DR arm.

Higher frequencies of drug-related AEs were observed in the 3DR arm than in the 2DR arm in this study, especially grade 1 or grade 2 dizziness. This finding was consistent with the toxicity profile of EFV, which includes neuropsychiatric AEs such as abnormal dreams, sleep disturbances, anxiety, depression, and dizziness [33, 34]. DTG + 3TC was well tolerated through 48 weeks with no additional or unexpected SAEs.

Drug induced liver injury (DILI) is a common and challenging AE in patients receiving EFV-based HAART regimens [35]. A total of 28.6% of participants with DILI had hepatocellular or mixed hepatocellular lesions (grade 1 or 2). Two patients withdrew from treatment because of grade 4 cholestasis liver injury. It had been reported that DTG results in increased levels of serum.

creatinine and moderate reductions in eGFR without changes in the iohexol-measured glomerular filtration rate [36]. In this study, changes in creatinine level at week 48 relative to baseline were more frequent in the 2DR arm. Given the higher rate of opportunistic infections in the 2DR arm, it seems possible that antifungal agents, especially amphotericin B because of its renal toxicity [37], were responsible for elevated creatinine levels. No significant differences between the 2DR and 3DR arms were observed in mean change from baseline in cystatin, urea, or eGFR at week 48.

There were several limitations to our study. This was an observational study with a limited sample size, which could lead to bias. In addition, the study enrolled predominantly men with a median age of 31 years. Because of the limited study duration, a roll-over study with 96-week and 144-week follow-up is ongoing to show the durable efficacy and safety of DTG + 3TC.

## Conclusions

Administration of DTG + 3TC achieved virologic suppression rapidly and with good immune reconstitution at weeks 12 and 24 in HAART-naive individuals, regardless of baseline viral load and with no CD4 cell count restrictions. DTG + 3TC could represent an optimal regimen for ART-naïve patients with advanced HIV-1 infection, especially those who require complex treatment plans or wish to minimize potential drug toxicity or DDIs.

## Data Availability

The datasets used or analysed during the current study available from the corresponding author on reasonable request.
